# Unravelling fetal enigmas: a case of suprasellar lesion

**DOI:** 10.1093/bjrcr/uaaf029

**Published:** 2025-05-14

**Authors:** Shravani Shinde, Dr Priscilla Joshi, Shriyash Pinglikar

**Affiliations:** Department of Radiodiagnosis, Bharati Hospitals, Bharati Vidyapeeth Deemed to be University, Pune, India; Department of Radiodiagnosis, Bharati Hospitals, Bharati Vidyapeeth Deemed to be University, Pune, India; Department of Radiodiagnosis, Bharati Hospitals, Bharati Vidyapeeth Deemed to be University, Pune, India

## Materials and methods

Fetal MRI following routine antenatal ultrasound (USG) was done.

## Instrumentation

1.5 T Philips Achieva System

GE Voluson E6

## Aims and objectives

This case highlights the vital role of advanced fetal imaging modalities, particularly fetal MRI, in the detailed assessment of rare intracranial abnormalities during pregnancy. Fetal MRI offers superior soft tissue contrast and multiplanar imaging capabilities, enabling more accurate diagnosis, informed prenatal counselling, and tailored perinatal management.

## Clinical presentation

A 32-year-old woman, gravida 2 para 1, at 28 weeks of gestation, presented for a routine antenatal ultrasound. Her medical history was unremarkable, and the current pregnancy had progressed without complications. First-trimester nuchal translucency (NT) screening and the second-trimester anomaly scan were both reported as normal, with all previous ultrasound evaluations showing no abnormalities.

However, during this routine scan, an unexpected finding emerged: a well-defined, solid-cystic lesion located in the suprasellar region, accompanied by mild dilatation of the lateral ventricles. The lesion measured approximately 3.6 × 3.6 cm, exhibited no significant vascularity on Doppler assessment, and showed no evidence of calcification. A prominently visualized cavum septum pellucidum (CSP) was also noted.

Fetal biometric measurements were within acceptable ranges: head circumference, abdominal circumference, and femur length corresponded to the 19.4th, 14.9th, and 29.6th percentiles, respectively, while the biparietal diameter was at the 67th percentile. The lateral ventricles were mildly prominent, measuring 10.8 mm and 11.1 mm. No spinal abnormalities were identified, and amniotic fluid volume was within normal limits.

Given the presence of this intracranial lesion, the patient was promptly referred for further evaluation with fetal MRI at our institution. She consented to the additional investigation and underwent fetal MRI the following day, allowing for more detailed characterization of the lesion and facilitating further diagnostic considerations.

## Ultrasound findings

**Figure 1.**
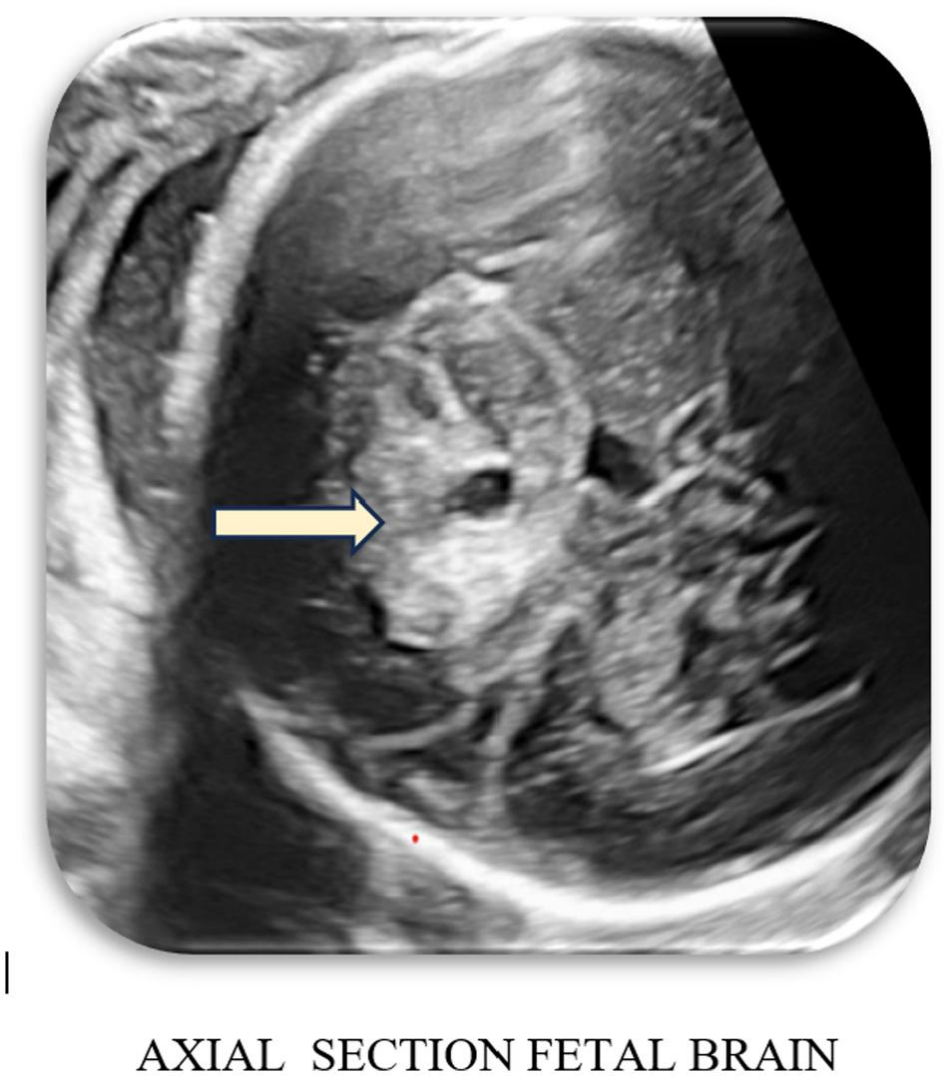
Heterogenous lesion with its epicentre in the midline in the region of the suprasellar cistern.

**Figure 2.**
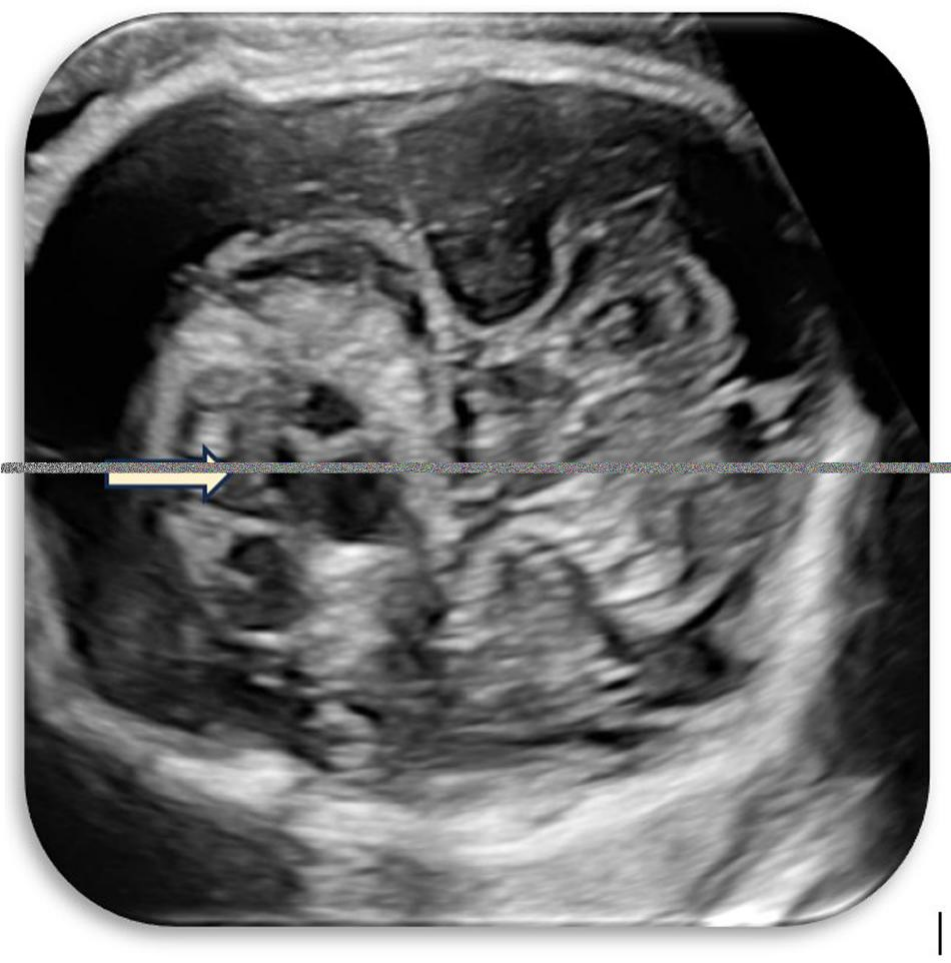
Solid and cystic areas within the lesion.

**Figure 3.**
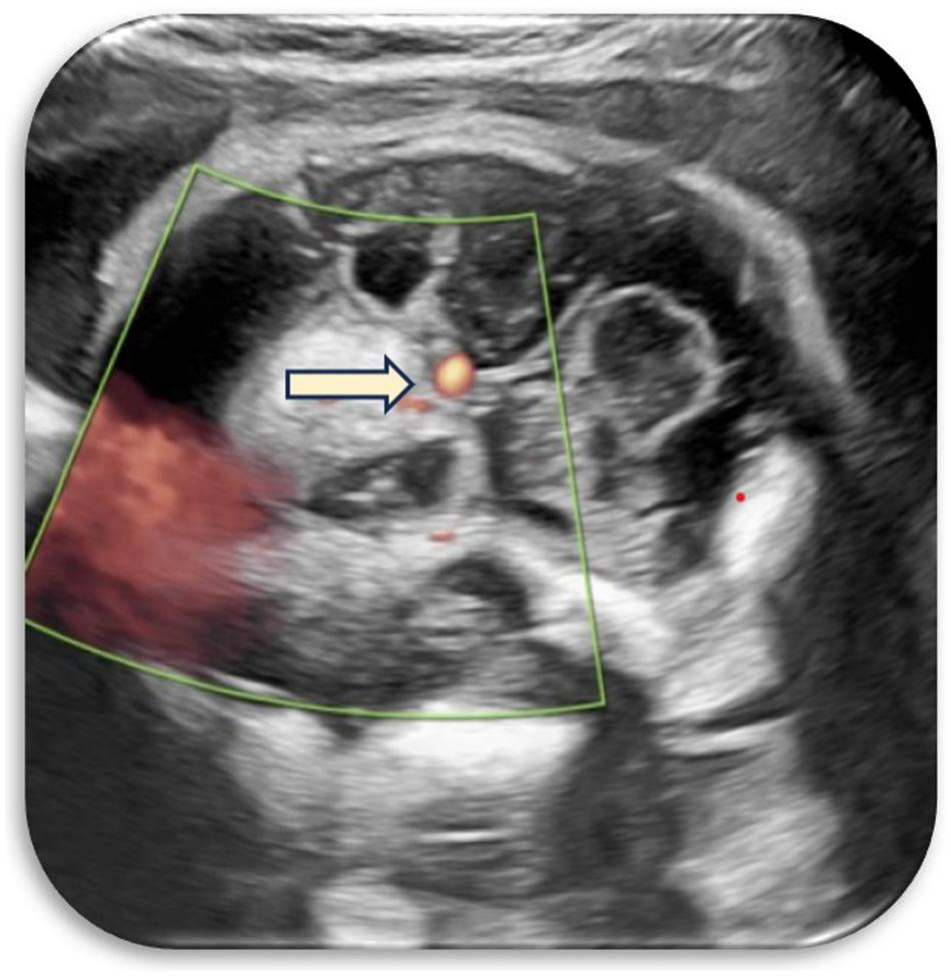
Mass seen abutting the internal carotid artery.

Initial ultrasound imaging revealed a heterogeneous suprasellar lesion with both solid and cystic components, centrally located in the suprasellar cistern. The lesion was seen abutting the internal carotid artery without evidence of calcification or significant vascularity on Doppler assessment. The CSP was prominently visualized and mild dilatation of the lateral ventricles was noted (Figures 1–3).

## Fetal MRI findings

**Figure 4.**
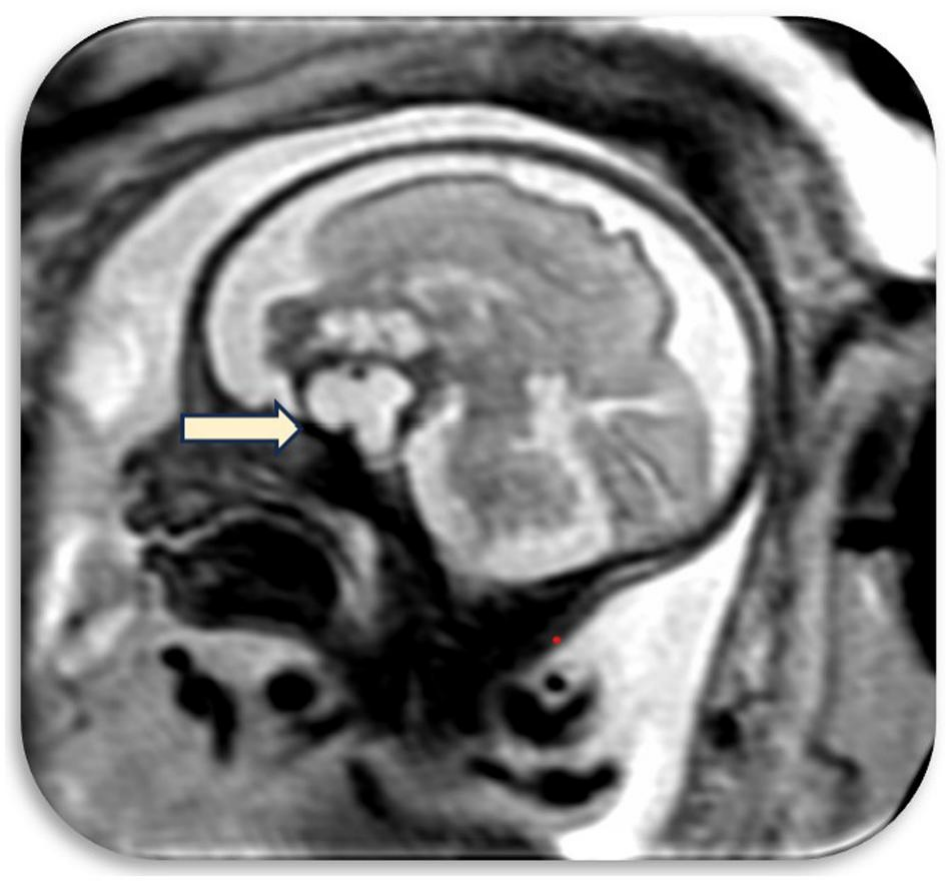
Anteriorly extension of the mass into the frontal region and posteriorly extension to the region of mamillary bodies.

A fetal MRI was performed using T1, T2, and BTFE sequences. On MRI imaging, A well-defined complex extra-axial mass was seen with its epicentre in the midline in the suprasellar region (Figure 4).

**Figure 5.**
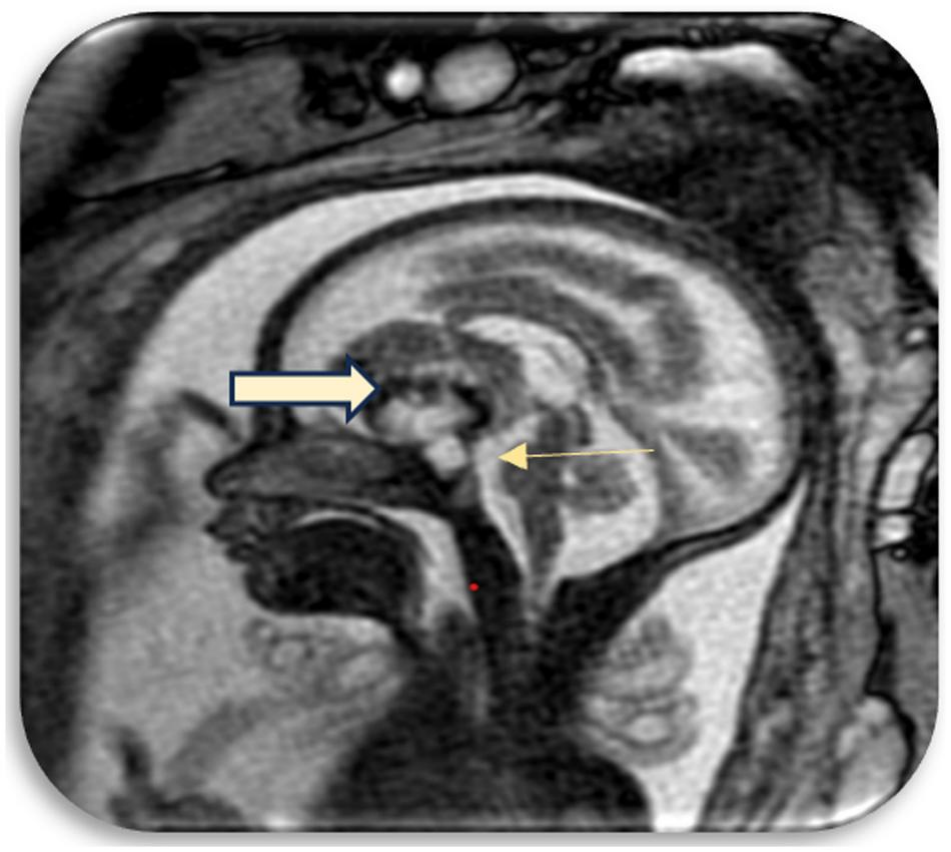
The pituitary gland compressed and caudally displaced by the mass.

Anteriorly, the mass was seen extending into the frontal region and posteriorly it was seen extending upto the region of mamillary bodies (Figure 5).

The pituitary gland was compressed and caudally displaced by the mass.

The lesion showed cystic and solid areas with hypointense areas predominantly in the periphery (Figures S1 and S2).

The mass compressed the posterior aspect of the third ventricle.

In this case, fetal MRI identified a well-defined suprasellar mass with both cystic and solid components, raising suspicion for craniopharyngioma, teratoma, or astrocytoma. Following multidisciplinary evaluation and parental counselling, the pregnancy was electively terminated. Histopathological analysis post-fetal autopsy confirmed the lesion to be an immature teratoma of the fetal brain, Grade I.

Teratomas of the central nervous system (CNS) are exceptionally rare, constituting less than 1% of all pediatric CNS tumours. When they do occur, they are most often located in the posterior fossa, particularly the cerebellum, though they can arise in other locations such as the pineal gland, spinal cord, or ventricles. Histologically, teratomas are classified as either mature, consisting of well-differentiated tissues, or immature, comprising primitive and undifferentiated elements. Immature teratomas tend to have a more aggressive growth pattern and heterogeneous imaging appearance, depending on the degree of differentiation.

On fetal MRI, immature teratomas typically present as complex, heterogeneous masses with cystic, solid, fat, and occasionally calcified components. On T1-weighted sequences, hyperintense areas correspond to fat, while solid portions demonstrate variable signal intensity. T2-weighted images often reveal hyperintense cystic areas and hypointense solid or calcified regions. Importantly, these lesions can exert mass effect, compressing adjacent brain structures and sometimes obstructing cerebrospinal fluid (CSF) pathways, leading to ventriculomegaly or fetal hydrops.

The differential diagnosis for suprasellar masses on fetal MRI is broad, encompassing benign, malignant, and developmental entities. These include:

Cystic lesions:

- Arachnoid cysts and colloid cysts: typically benign, fluid-filled lesions with no solid elements.
- Craniopharyngiomas: often exhibit both cystic and solid components, frequently with calcifications, and arise near the pituitary gland.

Neoplastic lesions:

- Optic pathway and hypothalamic gliomas: Usually seen in association with neurofibromatosis type 1; may present as irregular suprasellar masses.
- Astrocytomas: Can be located in various brain regions and show solid enhancement with variable imaging features.

Developmental anomalies:

- Pituitary hypoplasia or agenesis: may present as midline anomalies, often associated with endocrine dysfunction rather than mass effect.

Congenital malformations:

- Holoprosencephaly, Dandy-Walker malformation, and other midline anomalies may result in mass-like appearances in the suprasellar region.
- Hydrocephalus: secondary to mass-induced obstruction of CSF flow.

Infectious causes:

- Rare but important to consider; congenital infections such as toxoplasmosis may cause suprasellar involvement.

Imaging interpretation is crucial in differentiating among these possibilities. T1/T2-weighted sequences help delineate cystic versus solid components, while mass effect and anatomical distortion provide diagnostic clues. In postnatal imaging, contrast enhancement can aid in identifying vascular tumors, such as gliomas.

**Figure 6.**
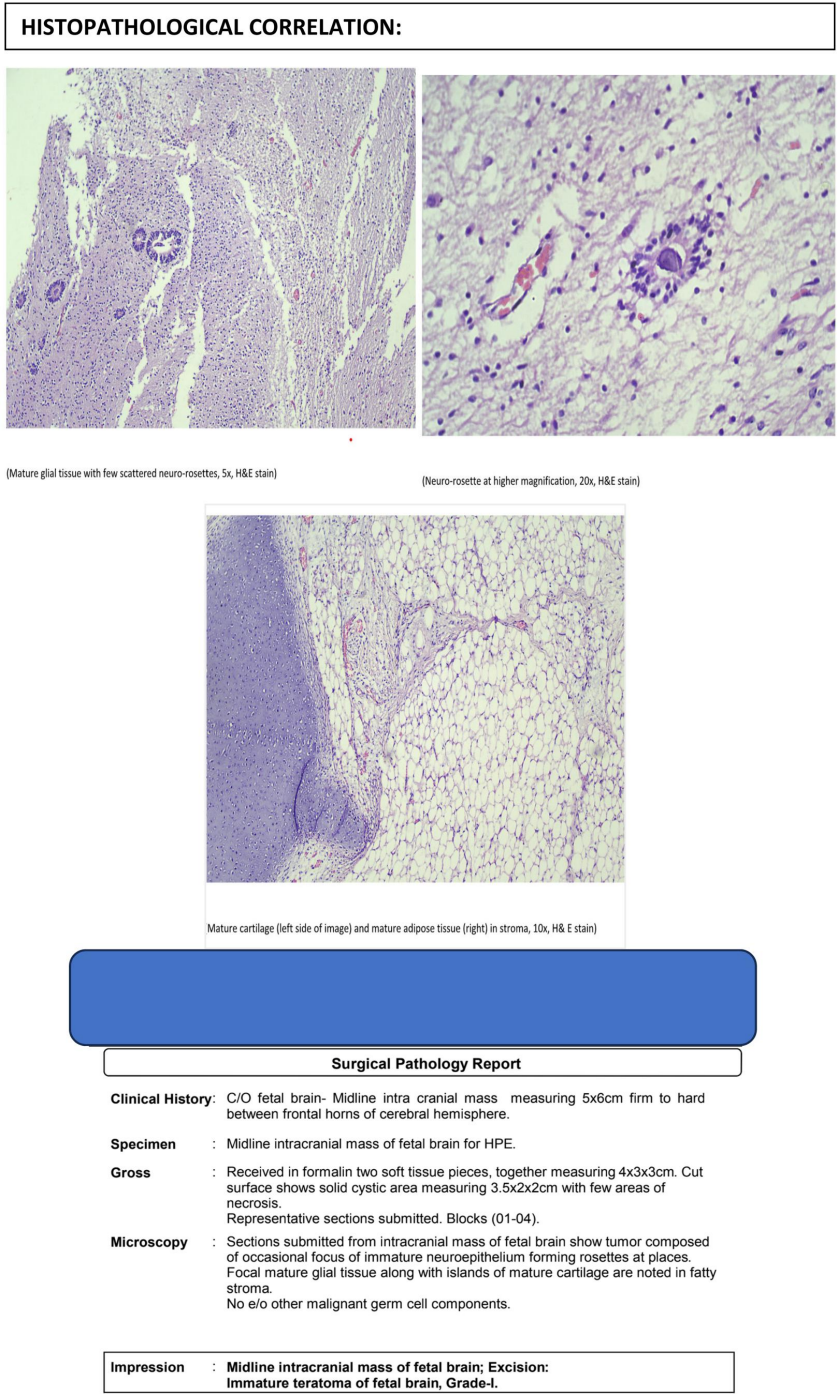
Histopathological analysis confirmed the diagnosis of an immature teratoma of the fetal brain, Grade I.

Given below are the classical imaging findings seen in craniopharyngiomas, teratomas, and astrocytomas (Figure 6).

Central nervous system teratomas in infants are extremely rare, comprising less than 1% of all paediatric CNS neoplasms. When they do occur, these tumours are most frequently located in the posterior fossa, particularly within the cerebellum, although they may also arise in the pineal region, spinal cord, or ventricular system.

Teratomas are histologically classified as either mature, composed of well-differentiated elements from all three germ layers, or immature, characterized by the presence of primitive, incompletely differentiated tissues. The degree of immaturity influences both the radiologic appearance and the biological behaviour of the tumour.

On fetal MRI, immature teratomas typically manifest as heterogeneous masses with both cystic and solid components. On T1-weighted sequences, fat-containing regions appear hyperintense, while solid components demonstrate variable signal intensity. On T2-weighted images, cystic areas are generally hyperintense, whereas solid or calcified regions may appear hypointense. These tumors can cause significant mass effect, resulting in compression of adjacent neural structures, and may occasionally be associated with fetal hydrops.

The radiological heterogeneity of immature teratomas reflects their variable tissue composition. Tumors with a higher degree of immaturity often exhibit reduced differentiation and a more disorganized architecture, which contributes to their complex imaging characteristics.

## Learning points

- Foetal MRI serves as an indispensable modality in the evaluation of intracranial tumours, offering unparalleled anatomical detail and tissue characterization that significantly surpass conventional prenatal imaging, thereby refining diagnostic accuracy.
- Timely detection and comprehensive assessment of these rare and complex lesions empower clinicians to strategize individualized perinatal management, including the anticipation of postnatal neurosurgical intervention, with the goal of optimizing neurological outcomes.
- A coordinated, multidisciplinary approach is paramount, uniting the expertise of obstetricians, radiologists, and paediatric neurosurgeons. Such collaboration ensures not only precise prenatal diagnosis and informed parental counselling but also seamless transition to postnatal care, ultimately enhancing both survival and quality of life for affected neonates.

## Conclusion

This case underscores the critical role of fetal MRI in the diagnosis and characterization of rare intracranial masses, enabling early and accurate identification, informed parental counselling, and coordinated management planning. In complex cases such as this, multidisciplinary collaboration between obstetricians, radiologists, and paediatric neurosurgeons is essential to optimize outcomes and guide decision-making regarding perinatal care and potential postnatal interventions.

## Supplementary Material

uaaf029_Supplementary_Data

## Figures and Tables

**Table 1.** Comparative radiological features of fetal craniopharyngioma, teratoma, and astrocytoma to aid in prenatal neuro-oncologic diagnosis.

Radiological finding	Fetal craniopharyngioma	Fetal teratoma	Fetal astrocytoma
Location	Often suprasellar, near the pituitary gland.	Location can vary (eg, brain, sacrococcygeal region).	Can occur in different brain regions (eg, cerebellum, cerebrum).
Shape/contour	Typically well-defined, may have cystic and solid components.	Variable, based on tissue types present.	Variable, based on grade and growth pattern.
Calcifications	Commonly seen as calcifications within the tumor.	May or may not have calcifications.	Less common, may be seen in higher-grade astrocytomas.
Cystic component	Common, with cysts of varying sizes.	Possible cystic areas, particularly in teratomas with diverse tissue types.	Cystic changes are less frequent; more often solid masses.
Solid component	Solid portions can be present, often in combination with cysts.	Solid components vary depending on teratoma type.	Solid masses are a characteristic feature.
Compression effects	Compression of adjacent structures (eg, optic pathways).	Can cause compression of neighbouring tissues.	Can compress surrounding brain tissue or ventricles.
Heterogeneity	Often heterogeneous appearance due to cystic and solid areas.	Heterogeneous appearance due to diverse tissue types.	Heterogeneous appearance due to variable cell density.
Cranial deformities	May cause distortion or enlargement of the sella turcica.	Uncommon unless the tumor is large and causes deformity.	Less likely to cause cranial deformities.
Malignancy features	Typically benign appearance, but local invasiveness can be seen.	Appearance may vary based on teratoma type; immature teratomas can appear more aggressive.	High-grade astrocytomas may show signs of malignancy like invasion or edema.
Ventricular effects	May cause hydrocephalus due to obstructed CSF pathways.	Uncommon to cause hydrocephalus unless large or obstructing ventricles.	Ventricular effects more common in certain locations or higher-grade tumors.

