# Supplementary figures and images for: Unravelling fetal enigmas: a case of suprasellar lesion
Source: BJR Case Rep. 2025 May 14;11(4):uaaf029. doi: 10.1093/bjrcr/uaaf029 (PMC12267136; doi:10.1093/bjrcr/uaaf029)

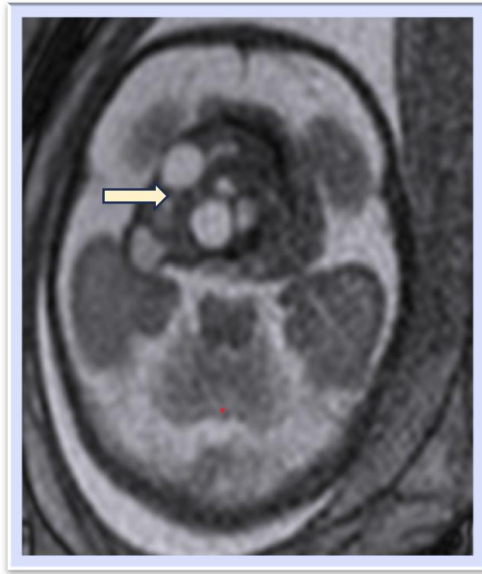

The lesion showed as well as cystic areas within

Supplement: uaaf029_Supplementary_Data [file uaaf029_supplementary_data.zip › uaaf029 Supplementary_Data/supplementary 2.1.pdf]

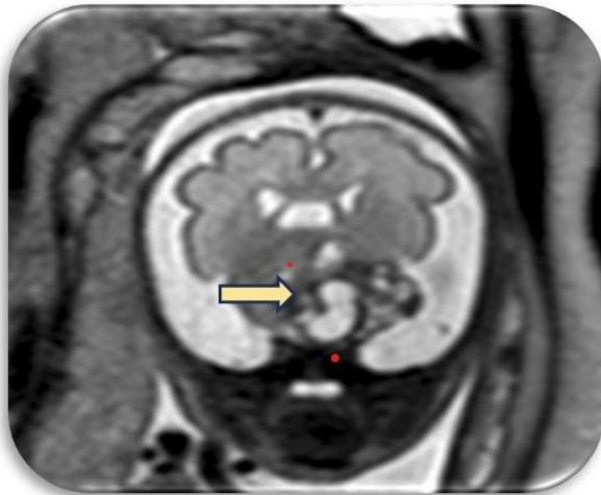

The mass compressed the posterior aspect of third ventricle

Supplement: uaaf029_Supplementary_Data [file uaaf029_supplementary_data.zip › uaaf029 Supplementary_Data/supplementary 2.2.pdf]
